# Editorial: Synergistic effects of antimicrobial peptides in overcoming resistance

**DOI:** 10.3389/fmicb.2024.1497614

**Published:** 2024-10-01

**Authors:** Corina Ciobanasu, Sónia Gonçalves Abreu, Berthony Deslouches

**Affiliations:** ^1^Department of Exact and Natural Sciences, Institute of Interdisciplinary Research, Alexandru I. Cuza University, Iasi, Romania; ^2^Instituto de Medicina Molecular, Faculdade de Medicina, Universidade de Lisboa, Lisbon, Portugal; ^3^Department of Environmental and Occupational Health, University of Pittsburgh School of Public Health, Pittsburgh, PA, United States

**Keywords:** antimicrobial peptides (AMP), antimicrobial synergy, antibiotic resistance, intracellular targeting, mechanism of AMPs

Antibiotic resistance (AMR) represents a growing global health threat which triggers demand for new antibiotics to combat resistant pathogens. Antimicrobial peptides (AMPs) are small (typically 10–50 amino acid residues in length), naturally occurring molecules that are part of the innate immune system across various organisms, including humans, animals, plants, and even microorganisms. In these organisms, AMPs play a crucial role in the body's defense against a wide range of pathogens such as bacteria, viruses or fungi. AMPs have gained attention as potential alternatives or adjuncts to traditional antibiotics, especially due to unique mechanisms of action that can target drug-resistant bacteria and their broad-spectrum activity. By leveraging their ability to disrupt bacterial membranes, inhibit biofilms, and modulate the immune response, AMPs can be developed into effective therapeutic agents. Combining AMPs with existing antibiotics, engineering them for greater stability and specificity is critical to overcoming current limitations and realizing the full potential of AMPs in combating AMR.

The Research Topic “*Synergistic effects of antimicrobial peptides in overcoming resistance*” cover research highlighting the critical issue of antimicrobial resistance and possible solutions in combating AMR, especially *Klebsiella pneumonia* and *Acinetobacter baumannii*.

The review by Taheri-Araghi covers *in vitro* and *in vivo* studies that analyze the synergy between AMPs and antibiotics. Combination therapies represent approaches against AMR, including both AMP-AMP and AMP-conventional antibiotic combinations. The efficacy of AMP-antibiotics can be influenced by several factors like concentrations or doses, the target organism, the presence and type of resistance mechanisms, and the local microenvironment. The optimization of therapy using AMPs and conventional antibiotics can be done by increasing the membrane permeability, direct enhancement of antibiotic potency, disruption of biofilms, and inhibition of resistance mechanisms, as *in vitro* studies revealed. *In vivo* studies, on the other hand, showed synergistic mechanisms not identifiable in *in vitro* studies such as the reduction of inflammation and immune modulation, delayed emergence of bacterial resistance and enhanced wound healing and tissue regeneration. However, to translate all these findings into effective therapies, extensive preclinical and clinical trials are required to confirm the safety, efficacy, and pharmacokinetics of these combination therapies in humans.

Brevicidine, a bacterial non-ribosomally produced cyclic lipopeptide displays synergistic effects in combination with conventional antibiotics like erythromycin, azithromycin, rifampicin, vancomycin, and meropenem against *Acinetobacter baumannii*, as Zhong et al. described in their article. The brevicidine–erythromycin combination exhibited potent antibacterial activity against *Acinetobacter baumannii* by enhancing the inhibition of adenosine triphosphate (ATP) biosynthesis and promoting the accumulation of reactive oxygen species (ROS), which are key mechanisms leading to bacterial cell death.

In another study, Wang et al. reevaluated the doses of polymyxin B against carbapenem-resistant gram-negative bacterial infection. Their study examined data from 77 patients with infections caused by carbapenem-resistant *Klebsiella pneumoniae*. Polymyxin B was significantly associated with clinical response, but the risk of nephrotoxicity was high. The authors concluded that empirical doses of 100 mg every 12 h, 1.25 mg/kg every 12 h for an 80 kg patient, or 1.5 mg/kg every 12 h for both 70 and 80 kg patients can provide effective therapeutic outcomes, but their efficacy must be weighed against the risk of nephrotoxicity.

Furthermore, the synergistic mechanisms underlying the combined efficacy of biological and chemical agents were discussed by Bi et al.. Pear black spot (PBS), which is caused by *Alternaria alternata*, results in significant damage in pears at global scale, reducing annual production by 20–50%, or even 80% in severe cases. Chemical control is still the main method used against pathogenic fungus *Alternaria alternata*. The combined use of safer chemical and microbial agents for controlling PBS is crucial for minimizing chemical pesticide use, reducing pollution, and enhancing ecological and environmental health. The experimental evidence showed that *Bacillus tequilensis 2_2a* exhibits a strong synergistic effect with difenoconazole, leading to hyphal entanglement, spore lysis, and the inhibition of PBS lesion formation *in vitro*.

Together, these research articles and reviews demonstrate the value of synergy between AMPs and antibiotics. Also, this Research Topic of articles provides an insightful examination of how antimicrobial peptides can be used to combat antibiotic resistance, a growing global health threat. The authors highlight the ability of AMPs to act in combination with commonly used antimicrobials already on the market, enhancing their efficacy through synergistic effects. AMPs have the potential to be a valuable tool in addressing actual AMR crisis, as the studies provide robust experimental evidence that demonstrates promising results for developing new and more effective treatment strategies.

